# Tuberculosis in developing countries: conditions for successful use of a decentralized approach in a rural health district

**DOI:** 10.11604/pamj.2014.17.198.3094

**Published:** 2014-03-13

**Authors:** Ziemlé Clément Méda, Chung-Chien Huang, Issiaka Sombié, Lassina Konaté, Paulin Küssome Somda, Arthur Diakourga Djibougou, Moussa Sanou

**Affiliations:** 1 Ministry of Health, Burkina Faso; 2 Health Care Administration Department, Taipei Medical University (TMU), Republic of China (Taiwan); 3 Municipal Taipei of Wan Fang Hospital, Republic of China (Taiwan); 4 Research Office of West African Health Organization (WAHO), Bobo Dioulasso, Burkina Faso; 5 National Institute of Health Sciences, Polytechnic University, Bobo Dioulasso, Burkina Faso

**Keywords:** Developing countries, screening program, detection, HIV co-infection, tuberculosis, rural district

## Abstract

**Introduction:**

This article reports the results and the lessons learned from implementing the decentralized approach to tuberculosis (TB) detection and treatment, embedded with Human Immunodeficiency Virus (HIV) co-infection in health district. The objective was to increase the TB screening indicators in the district using the common ways for offering care to patients in health district.

**Methods:**

Conducted from August 2006 to July 2007, this large-scale intervention using Non-experimental study Designs has implemented a decentralized approach for fighting against TB in Orodara Health District (OHD), Burkina Faso. Pretest-posttest design has been used for quantitative part using indicators in one hand, and postests-only design for the qualitative part in other hand. In the pretest-posttest design, the TB indicators from years before 2006 (from 2002 to 2005) were used as earlier measurement observations allowing examining changes over time. The decentralized approach was incorporated into the annual planning of the OHD. For the quantitative study design, indicators used were those from National TB Program in Burkina Faso: TB detection rate, incidence density of TB per 100,000 inhabitants per year, and HIV prevalence in incident TB cases with positive smears. Data entry and analysis employed Microsoft Access and Excel software. For the qualitative, in-depth interview was used in which a total of 16 persons have been interviewed. Discussions were tape-recorded and transcribed verbatim for analysis using the computer-based qualitative software program named QSR NVIVO

**Results:**

There were a total of 99,259 outpatient visits during the study period: the7,345 patients (7.43%) presented with cough. Of the 7,345 patient having cough, 503 cases (6.8%) were declared chronic coughing. These 503 patients were screened for TB, including 35.59% whose coughing had lasted 10 to 15 days. We observed an increase in a measured variable was observed. The TB detection rate and incidence-density rate based on positive smears were 16.11% (11.00% in 2005) and 10.42 per 100,000 inhabitants per year (6.88 per 100,000 inhabitants in 2005), respectively. There were 29 patients positive for TB: 41.37% of these had cough lasting 10 to 15 days, 10.34% were also positive for HIV, and 68.97% were from rural areas. Health workers and patients reported satisfaction with the intervention. It was found that implementing a decentralized approach to TB prevention in rural areas is plausible and effective under some conditions: considering that health district system is functional; carefully designing the intervention for TB case management; setting up and implementing of decentralized approach including strong monitoring; and taking into account the all financing, community and volunteer involvement, evaluation of the cost savings from integrating specific donor funding, and being supported by regional and central levels including National TB program.

**Conclusion:**

The study has shown that TB detection rate can be increased by implementing a decentralized approach to primary care. When carefully implemented, a decentralized approach is a suitable approach to TB and HIV prevention in rural and inaccessible settings.

## Introduction

Direct Observation of Treatment Short-course (DOTS) strategy to disease prevention employs tuberculosis (TB) treatment unit as the detection unit. The main difficulties of implementing DOTS for fighting against TB in developing countries are a lack of assiduity among health personnel, the daily commute, long distances, high transportation costs and the high number of pills to be consumed [1]. In other words, the main limitation of DOTS strategy, only possible in the treatment unit, is its centralized nature [2]. There were two TB centers in the Orodara Health District (OHD) of Burkina Faso: Orodara District Hospital and the N'Dorola peripheral health center.

TB has re-emerged as a worldwide health problem since 1986; and TB counts among the most important public health priorities, due to its gravity, its magnitude and its burden on socio-economic wellbeing in many countries [3]. The number of TB cases is increasing in association with Human Immunodeficiency Virus (HIV), and HIV prevalence among TB patients in Burkina Faso is estimated at 35.2% [4]. Thus there were many unexplored potential synergies between TB and HIV/AIDS program objectives and activities [5].

TB is a great issue in developing countries, since the DOTS for managing TB cases requires an efficient health system [6, 7], and orderly organization adapted to specific conditions [7, 8]. Decentralization offers a useful alternative strategy and a real opportunity for progress in these contexts by reducing the number of health encounters and travel distance required of patients, eliminating the need for referral, and moving TB treatment and screening closer to the population [9]. Indeed, distance and travel costs for patients going to TB centers are negative factors for most TB programs, as they are associated with delays in seeking diagnosis or treatment of tuberculosis [10]. Decentralizing tuberculosis case management is a proven, satisfactory strategy that offers effective solutions as already tested in eight pilot peripheral health centers; there is still a need for more research to capitalize this experiment [7].

This article reports the results and the lessons learned from implementing the decentralized approach to TB detection and treatment (embedded with HIV detection) in all 40 peripheral health centers within OHD, one of the inaccessible rural districts of Burkina Faso.

## Methods

**Study setting:** the study was carried out in Burkina Faso, where the DOTS is implemented in all health districts, and in regional and national hospitals. Located in the West of Burkina Faso, the OHD is comprised of Oradora District Hospital and 40 peripheral health centers; there were no private health services. The district covers a large area of 8,307 square kilometers with a population 2006 of 261,710. In 2006, only 38% of the OHD had connecting roads, and an uneven access to health centers [11]. The TB detection rate was 10.29% in 2005; the situation was worse in rural areas. Sputum examinations and HIV tests were only possible in the Oradora District Hospital and the N'Dorola health center. There were no rapid tests for HIV screening in the OHD at the beginning of the intervention, and no radiology service was available in the Oradora District Hospital.

### Operational definitions

The study focused on pulmonary TB which is the most common clinical form, appearing in 80% to 91% of all TB cases [12, 13]. In Burkina Faso, pulmonary TB cases marked by a positive smear test were 74.33% [12]. Chronic cough was chosen as the most physiolgical functioning symptom [14]; defined chronic cough as coughing lasting ten days or more. TB smear result was considered positive when at least two sputum samples from the patient were examined and the TB germ was detected by optic microscopy after Zielh-Nelsen coloration.

### The intervention

**Figure 1:**
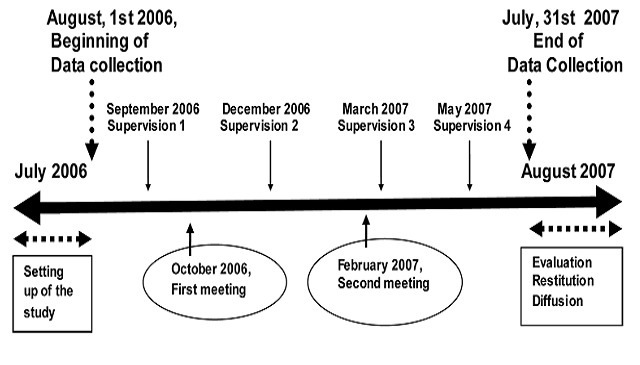
The Iintervention planning during the decentralized approach

**Figure 2:**
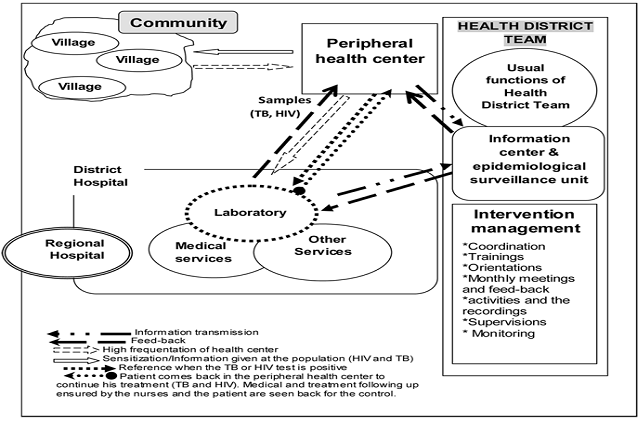
The Iintervention planning during the decentralized approach

Conducted from August 2006 to July 2007, this Non-experimental study Designs [15] was an intervention that implemented a decentralized approach for fighting against TB. Pretest-posttest design has been used for quantitative part using indicators in one hand, and postests-only design for the qualitative part in other hand. In the pretest-posttest design, the TB indicators from years before 2006 (from 2002 to 2005) were used as earlier measurement observations allowing to examine changes over time. The decentralized approach was incorporated into the annual planning of the OHD (Figure 1, Figure 2) and the project was coordinated and supervised by the Health District Team (HDT) (Figure 2). The objective was to increase the TB screening indicators in the district using the common ways for offering care to patients in health district. Thus, the intervention was implemented as a complementary strategy of the regular activities of the Health District Team (HDT) following a research-action track.

This project targeted adults aged 15 and older in the population at large who experienced coughing that had started at least ten days before, patients known to have pulmonary TB, and patients who had positive smear test restults but were not under anti-tuberculosis treatment yet.

The setting up of the intervention started by assembling the study team (HTD), study documents and material for samples in all health centers, and TB health education sessions per village in each peripheral health center by trained health workers. About 90% of health workers received training on TB management: about TB case definition, diagnosis and treatment of TB and HIV infections, and how to take sputum samples according to the national guidelines.

Data were collected from August, 1^st^ 2006 to July, 31^st^ 2007. In the same period there were the supervisory visits and meetings with health workers for feedback and re-orientation (Figure 1). Each health center recruited patients during consultations based on the inclusion criteria and with informed consent of the patients. Sputum samples where collected from patients complained of chronic cough; after coding, the sputum boxes were sent to the laboratory weekly through the routine transmission of case notification (Figure 2). Then, the laboratory communicated the results of TB tests to the health center and to the information office. Each case was registered in the medical charts of the health facilities and in the laboratory register. When a smear was positive for TB, the health center sent a second sample for HIV testing following counseling. The information transmission and verification process for HIV was the same as that for TB. HIV counseling was done before giving the final HIV results to the patient.

For follow-up and treatment, patients were included in the TB and/or HIV patients cohort of the Hospital District respecting ethical aspects according to the national guidelines. Then, the patient was transferred to his/her health center. In the health center, the nurses gave the anti-tuberculosis treatment to the patient under DOTS, and reminded the patients about the next appointment at Orodara for the clinical and biological follow-up. Purchasing and restocking of laboratory and TB treatment materials and quality control were carried out as usual by the national TB program.

The final part of this study was completed by interviewing actors within the health district system (both in the health system and in the community) and the feedback of the results to the actors.

### Indicators and statistical analysis

**Figure 3:**
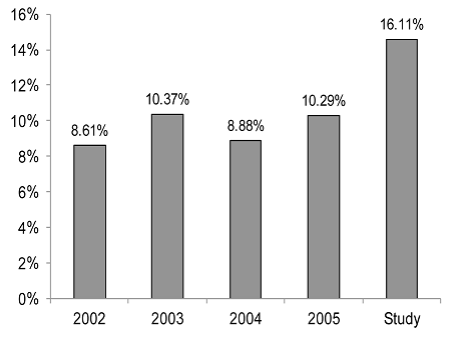
Comparison of TB detection rate according to the National TB Program drom 2002 to 2005 and the study in Orodara Health District, Burkina Faso

**Figure 4:**
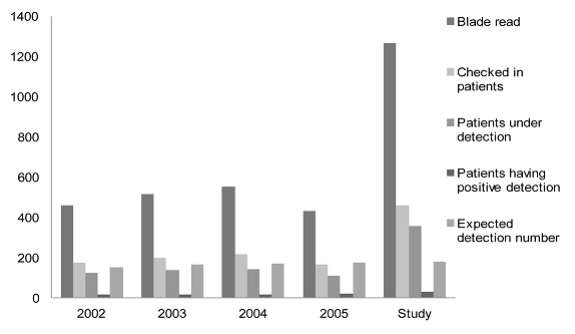
Comparison of the TB indicators reporting according to the National TB program from 2002 to 2005 and the study in Orodara Health District, Burkina Faso

Indicators used were those from National TB Program in Burkina Faso. TB detection rate was the total TB new cases (newly diagnosed) in the OHD divided by the total TB suspect cases for one year and multiplied by 100 [7]. The incidence density of TB per 100,000 inhabitants per year was the number of new TB cases with positive smears divided by the total population 2007, multiplied by 100,000 for the study period. HIV prevalence was also calculated in incident TB cases with positive smears. The indicators produced by the study were compared to those before the intervention (Figure 3, Figure 4). Data entry and analysis employed Microsoft Access and Excel software.

### The qualitative study design

In-depth interview was used for this qualitative study, in which a total of 16 persons have been interviewed: the Head Medical Doctor of the HD (Health District manager), five (05) health workers and ten (10) TB patients by convenient method after informed consent. Patients were recruited with the help from health workers who informed the patients and negotiated the appointment time. Another appointment was negotiated for the interview. The place of the interview was TB detection and treatment Center (TDTC) of Oradora District Hospital. The individual interviews were conducted to emphasize relevant questions related to access to care and treatment, the pertinence of the intervention, and benefits for health system and TB patients. The moderator was fluent in *French* and *Dioula* languages, and filled the discussion guide. To minimize ‘social desirability’[16], participants were reminded that the facilitators had no association with the health center. Discussions were tape-recorded and transcribed verbatim for analysis using the computer-based qualitative software program.

### Study financing

This intervention was funded by the OHD budget (about 40%) and with the support of a Non-Governmental Organization (NGO) called Medicus Mundis Castilla La Mancha (about 60%).

### Research approval

This study was approved by the Institutional Review Board (IRB) of the Hauts-Bassins Regional Health Direction, in Burkina Faso. To get an external judgment on the procedures and considering the way for approving and validating all activities of Health District by the national and regional levels, the intervention was embedded to the annual plans 2006 and 2007 of the Orodara Health District (OHD). From this perspective, the HDT presented its annual planning 2006 and 2007 and got the approval from the technical and financial session of the Hauts-Bassins region in January 2006; every year, each session is attended by stakeholders from health system from national, regional and health district levels, as well as civil society and administration representatives.

During the intervention, patients who verbally consented after being individually informed about the study were admitted. The informed consent from caretakers was obtained, as well as their commitment to respect confidentiality of the data and all information revealed. Additionally, the informed consent from guardians on the behalf of the minors/children participants was not involved in this study. the study has not included patients aged less than 15 years old. The IRB approved the consent from patients aged between 15 to 18 years old. Patients aged between 15 to 18 years old were considered as those at least having 18 years old according to the national guidelines in the regular medical consultation.

About the verbal informed consent and its use, the health workers presented to the participants the purpose of the research. They verified that patients understood and chose to undergo a given procedure; and the participation was voluntary and involved an informal interview lasting between thirty minutes and an hour. Additionally, they showed that this research has no known risks, and it protected the participant privacy. Participant identity or personal information has not been disclosed in any publication that may result from the study.

For followup and suitable treatment, patients were included in the TB and/or HIV patients′ cohort of the district hospital respecting ethical aspects according to the national guidelines.

## Results

A total of 422 TB health education sessions were carried out representing an average of 10.6 sessions per peripheral health center, and constituting a rate of 95.91%. About 29 supervisory visits were performed by the Health Distrit Team: 7 quaterly sessions with 5-6 peripheral health centers included in one session. The health district held two meetings at its headquarter. A total of 99,259 patients sought care through the health centers: 7,345 (7.4%) complained of cough (monthly average between 3.6% and 10.4%); and 91.5% of the total number of patients were from rural areas.

Of the 7,345 coughing patients, 503 patients (6.8%) were declared having chronic cough: 448 of these (89.1%) were from rural areas; 179 (35.6%) had experienced cough over a period of 10 to 15 days; and their mean age was 23.4 years old (22.0-24.8). Males constituted 58% of the 503 chronic coughing patients with a mean age of 22.0 (21.0 - 24.0). There was a difference of the mean age between females and males (p= 0.037). Among the patients who underwent sputum exams, 96.22% did not have a formal salary. The health centers collected a monthly average of 42 cases and sent their samples to the laboratory. Of the 503 chronic coughing cases who received further TB diagnosis, the TB cases identified in the study period was 30, of which 29 had positive smear test results. The true TB positivity rate was 5.8% (4.2%-7.0%). The proportion of patients with positive smear results and coughing duration of 15 days or more was 6.7% (0.04%-9.40%). The rate of TB cases identified by positive smear results that originated from rural areas or lived further than 10 kilometers from a health center was 68.97%. Of the 29 patients positive for TB, 12 (41.4%) had been coughing between 10 and 15 days; 18 (62.1%) were from rural settings; and three (10.3%) were HIV-positive (2 HIV type 1 and 1 HIV type 2).

There was no difference between the duration of coughing and TB test results (p= 0.827). There was a difference between gender and TB test results (p= 0.009). And there was no difference between HIV test results and TB test results (p= 0.807). The TB detection rate was 16.11%, and had been 11% for previous years (Figure 3, Figure 4). The annual TB incidence density rate identified through positive smear results was 10.4 per 100,000 inhabitants (with 13.2 per 100,000 in urban settings and 10.2 per 100,000 in rural settings) and was 6.88 per 100,000 inhabitants per year in 2005. The HIV-positive patients detected were included in the HIV file according to guidelines; then patients with HIV type 2 we referred to the National Teaching Hospital of Bobo Dioulasso.

Assessing the impact, there were many benefits for the health system and communities in the Orodara Health District. It was found that implementing a decentralized approach to TB prevention in rural areas is plausible and effective under some conditions (Appendix 1). Health workers and patients reported satisfaction with the intervention. In summary, the intervention contributed to achieve the objectives of the health district, impacted positively the workforce cohesion, gave a good feed-back form the population, and increased the attendance of the health centers. The HDM said that *“Considering it and the insufficient geographical accessibility of the health services in the health district, we initiated this intervention financed by the NGO Medicus Mundi Castilla La Mancha (MMCLM) and ourselves?. So everyhing is possible, it must just be the vision, the willingness to get organized in function. I am very glad because the people in my health district have been ranked as having the second best health in my country and the best health status in our region*.” As indicated by the HDM, the TB indicators are main clinical criteria for annual apraival of health districts at the regional and national levels. Still from health workers, one said that *"Before I worked without considering certains things. For instance, I was waiting for the patients, whether they came or not, that was not my concern. Also, a cough is a cough, only I knew it needs antibiotics and often that is good enough. But I recognized some errors because certain patients kept coming back. There was the case of a patient I saw one month and half back. Using this approach of the project, we found out it is TB. Now, I understand many more things in my job: I have less problems with my chief and I see the impact of sensitization and the purpose of our writing in the registers and the recording. Previously, I did the recording merely because the superiors required this. With this project, the patients come more, and I think it is better for us, because we are more considered for it". Another added that “Due to my chief and considering the statistical data, we have been convinced to do something for the patients, because certains indicators were too low, like TB testing rates. Thus, we were neglecting to report the cases in general, primarily chronic cough. This project has helped us so much. Now, my relationship with my colleagues is much more cohesive because they understand the importance of what we are doing. Also, the entire team is glad because the population of our health center has found this approach exceptionally good. We have raised the number of persons who are in curative consultation”*.

Additionally, the project received a positive reaction from the patients mainly about the access to care. It appears that the program has succeeded in fostering wider geographic and financial access to care, and in bringing care to patients in their everyday environment. One TB patient declared that *“At the onset, I did not understand an important thing about my health because I went to a traditional practitioner. Then, I learned information about sensitization. One brother told me of this with some TB signs. So, I came in to the health center. It is why I arrived in the health center. And I am glad because all information concerning my health is in one place. God Thank you”*.? Another TB patient stated that *“I coughed and it did not finish. After one month, I came to the peripeheral health center. Then, the health worker explained to me that it could be TB and it is necessary to have some health exams from Orodara. Immediately, I would want to come back, because, I had a bad experience of traveling to Oradara for health care. I lost my all money seeking healing in this time. And you can go for one day and it becomes two or three days. The health worker assured me that I would not have to go to Orodara myself. So I am glad I do not move, I did not have to travel to Orodara. Now, I am under treatment and it is like I do not spend anything. And, I continue working without a problem”*. A third said that *“For once, we get cared for in place in our peripheral health center. Before, my neighbour died because he was not able to go to Orodara to get his medication. I am glad because it is unnecessary now to travel and you can work and do not disturb anybody. Otherwise, it was inconvenient seeking somebody to accompany me to Orodara for long treatments.”*

Above all, for the health system, this helped to generate public enthusiasm for the health workers, facilitating better relationships between patients and health workers, and contributing to reaching the objectives.

## Discussion

Applying a decentralized approach to health care delivery, the results showed an increase of TB detection rate and improved HIV detection among TB incident cases. The study tackled equity issue in distribution of public health resources between urban and rural districts. Despite these positive results and significant contribution to public health practices, the study had some unavoidable methodological limitations. For example, there was no health district control, even whether the intervention was about all the 40 peripheral health centers of the health district. Indeed, Likewise, there was no cost-effectiveness assessment of the intervention, even though there was substantial benefit for the patients.

### TB detection and access to care

The results of this study were in comformity with previous ones with the increase of TB detection rate [7]. The screening of entrants is particularly important to avoid missed smear-positive TB cases [17]. The priorities of fighting TB are diagnosis and treatment of TB carriers.

Prior studies showed the importance of geographic and financial access to care for evaluating the achievements of TB programs [9, 18, 19], and have promoted the decentralization of services [20]. Similarly, Sanou and colleagues pointed out that barriers including geography, poverty and gender are also significant [2]. Moreover, environmental issues that negatively affected TB patients related to sense of safety, home environment, transportation and financial security [21]. During their interviews, patients and health workers in the study confirmed the importance of these factors.

### Decentralized approach, as a public health strategy in some contexts

The decentralized approach to health care delivery provides an opportunity to improve services in resource-scarce settings. Indeed, decentralization to rural health facilities of follow-up for anti-retroviral treatment (ART) using an integrated primary care system appears to offer a safe and effective way to rapidly scale-up ART and improves both geographic equity in access to HIV-related services and adherence to ART [22].

Policy-makers and health leaders need to find innovative solutions to the problem of escalating health care costs that go beyond limited resources, and shifting budgets among the government, individuals, and business [23]. Decentralized approach to care is a useful strategy in this context. Incorporated into the OHD planning and adapted to the context, the present intervention contributed to reducing the inequality between rural and urban areas in terms of health facilities and access to care.

To succeed in decentralizing preventive medicine, including TB case management, it is important to prudently design the intervention and to consider the key steps for decentralizing TB care management at the peripheral health center level (Appendix 1). This listing of key steps for decentralizing TB care management was based on lessons learned from the present study and literature review [9, 16, 17, 20, 22–25]. These keys steps reflect the functional health system itself, the conception of intervention used for TB case management, the setting up and implementation of a decentralized approach that includes efficacious monitoring, and other considerations. The intervention protocol required improving patient counseling and communication, decentralization of treatment, enabling patient choice of DOT supporter, and reinforcement of supervisory activities; these measures led to improvement in patient outcomes compared with the usual TB control procedures that were in place [25].

Decentralization of services enables fulfillment of two recommendations: issuing a ‘Stop TB’ call communicating the goal of reducing the TB burden, and health system strengthening based upon primary health care and engaging all care providers [24]. More importantly, decentralization may reduce congestion at larger hospitals creating further opportunity to improve quality of care [9, 18]. However when decentralizing services, it is essential to establish procedures for facilitating evaluation and to identify and solve problems amenable to correction [26]. Finally, decentralizing TB treatment produced good treatment outcomes, can reduce expenditures by health providers, patients and families, and improve cost-effectiveness [19]. In this study, implementing a decentralized TB treatment and screening program in the OHD of Burkina Faso produced an increase of TB screening and detection rates, increased the satisfaction of both health workers and patients, and facilitated greater equity in access to care.

## Conclusion

Decentralization of care improved TB detection at the district level by actively involving staff of health centers through a primary care model: adapted for managing TB and HIV problems, overcoming physical and financial barriers to care, and lack of information experienced by patients, in order to accomplish public health objectives for communities. Moreover, decentralization is helpful for fighting TB and HIV in expansive rural areas before the establishment of the HIV rapid testing. TB detection through positive smear test results remains the best way to eliminate TB carriers in the early stages of the disease and thus to stop TB dissemination. It must be reviewed that the operational definition of chronic cough by considering chronic cough as coughing lasting ten days or more.

## References

[1] Ouedraogo M, Kouanda S, Dembele M, Ouedraogo SM (2006). Obstacles to the implementation of directly observed treatment in Ouagadougou, Burkina Faso. Int J Tuber Lung Dis..

[2] Sanou A, Dembele M, Theobald S, Macq J (2004). Access and adhering to tuberculosis treatment: barriers faced by patients and communities in Burkina Faso. Int J Tuber Lung Dis..

[3] Girard PM, Katlama CH, Pialoux G (1998). Tuberculose et Sida. Edition Doin Paris..

[4] Ministère de la santé (2002). Statistiques du Programme National de Lutte contre la tuberculose (PNT).

[5] WHO (2003). Guidelines for implementing collaborative TB and HIV programme activities.

[6] Mahendradhata Y, Lambert ML, Van Deun A, Matthys F (2003). Strong general health care system: a prerequisite to reach global tuberculosis control targets. Int J Health Plann Manage..

[7] Drabo KM, Dauby C, Coste T, Dembele M, Hien C, Ouedraogo A (2006). Decentralising tuberculosis case management in two districts of Burkina Faso. Int J Tuberc Lung Dis..

[8] Macq JC, Theobold S, Dick J, Dembele M (2003). An exploration of the concept of directly observed treatment (DOT) for tuberculosis patients: from a uniform to a customised approach. Int J Tuberc Lung Dis..

[9] Needham D, Bowman D, Foster S, Godfrey-Faussett P (2004). Patient care seeking barriers and tuberculosis programme reform: a qualitative study. Health Policy..

[10] Saint S, Ikushi O, Nobukatsu I (2006). Decentralized DOTS shortens delay to TB treatment significantly in Cambodia. Kekkaku.

[11] Orodara Health district (2005). Annual planning of Orodara Health district 2006.

[12] Ouedraogo M, Ouedraogo G, Ouedraogo SM, Zigani A, Bambara M, Somé L (1999). A propos de la tuberculose a Ouagadougou: Etude retrospective a propos de 2 202 cas. Médecine d Afrique Noire..

[13] LeBeau (1994). Pneumologie francophone. Ellipse, Paris.

[14] Revue des Maladies Respiratoires (2003). Diagnostic clinique et bactériologique de la tuberculose. Revue des Maladies Respiratoires.

[15] Andrew Fisher J. Foreit (2002). Designing a Study Intervention, Study Designs. From Designing HIV/AIDS Intervention Studies: An Operations Research Handbook.

[16] Shrestha-Kuwahara R, Wilce M, DeLuca N, Taylor Z (2003). Factors associated with identifying tuberculosis contacts. Int J Tuberc Lung Dis..

[17] Carbonara S, Babudieri S, Longo B, Starnini G, Monarca R, Brunetti B (2005). Correlates of Mycobacterium tuberculosis infection in a prison population. Eur Respir J..

[18] Needham DM, Foster SD, Tomlinson G, Godfrey-Faussett P (2001). Socio-economic, gender and health services factors affecting diagnostic delay for tuberculosis patients in urban Zambia. Tropical Medicine and International Health..

[19] Nyirenda TE, Kwanjana J, Gondwe M, Salaniponi FML Whence decentralized TB treatment and whither TB program in Malawi?. Malawi Medical Journal, Viewpoint: 21-23.

[20] Demissie M, Lindtjorn B, Berhane Y (2002). Patient and health service delay in the diagnosis of pulmonary tuberculosis in Ethiopia. BMC Public Health.

[21] Dhuria M, Sharma N, Ingle GK (2008). Impact of tuberculosis on the quality of life. Indian J Community Med.

[22] Chan A (2010). Outcome assessment of decentralization of antiretroviral therapyprovision in a rural district of Malawi using an integrated primary care model. Tropical Medecine and International Health.

[23] Foege WH, Nils D, Black RE, Perason CE (2005). Global health leadership and management.

[24] WHO Report 2009: Global tuberculosis control - epidemiology, strategy, financing. http://www.who.int/tb/publications/global_report/2009/en/index.html.

[25] Thiam S, Lefevre AM, Ndiaye A, Ba F, Fielding KI, Ndir M (2007). Effectiveness of a Strategy to Improve Adherence to Tuberculosis Treatment in a Resource-Poor Setting. A Cluster Randomized Controlled Trial. JAMA.

[26] Arnadottir TH, Phongosa B, Chittamany P, Soukaseum H (2002). Decentralizing tuberculosis treatment: follow-up of patients during the transitional period. Int J Tuberc Lung Dis..

